# Herd-Level Prevalence of High Fat-to-Protein Ratio and Associated Factors During Early Lactation in Irish Spring-Calving Dairy Herds

**DOI:** 10.3390/ani15213068

**Published:** 2025-10-23

**Authors:** Raffaela Marian, Conor G. McAloon, Emmet T. Kelly, Catherine I. McAloon, Finbar J. Mulligan, Luke O’Grady, Marijke Beltman, Eoin G. Ryan

**Affiliations:** 1 Section of Herd Health and Animal Husbandry, School of Veterinary Medicine, University College Dublin, Belfield, D04 W6F6 Dublin 4, Ireland; 2 Centre for Veterinary Epidemiology and Risk Analysis, School of Veterinary Medicine, University College Dublin, Belfield, D04 W6F6 Dublin 4, Ireland

**Keywords:** fat-to-protein ratio, seasonal calving system, negative energy balance

## Abstract

**Simple Summary:**

This study used national milk data from over 11,051 Irish spring-calving dairy herds from 2014 to 2023 to assess herd-level prevalence of high fat-to-protein ratio (FPR) (>1.4), as a proxy measure for negative energy balance (NEB), and associated factors. NEB during early lactation increases the risk of metabolic disease, reduces fertility performance, and limits profitability in dairy systems. Median herd-level prevalence of high FPR (>1.4) <30 DIM had a 10-year mean of 14.57% (annual median ranged from 10.81 to 20%), decreasing to 8.10% (annual median ranged from 6.09 to 12.90%) from ≥30 to <60 DIM, with annual variation noted. Results highlighted seasonal and herd-level factors influencing herd-level prevalence of high FPR. The months of February and March, coinciding with housing, feeding of conserved forage, peak calving, and early grazing periods, were associated with the highest prevalence. Genetic traits and 305-day milk yield were linked to herd-level prevalence of high FPR. Higher-yielding herds were associated with reduced odds of increased herd-level prevalence of high FPR, i.e., reduced risk of NEB at herd level. These findings identify important factors associated with herd-level prevalence of high FPR during early lactation, which can help inform future preventative strategies aimed at improving herd health, productivity, and sustainability in Irish dairy herds.

**Abstract:**

High fat-to-protein ratio (FPR) has been used as a proxy measure for negative energy balance (NEB) in dairy herds. This study describes herd-level prevalence of high FPR during early lactation in Irish spring-calving dairy herds and associated risk factors. A retrospective observational study was conducted using 10 years (2014–2023) of national milk recording data from 11,051 unique herds. Median herd-level prevalence of high FPR (>1.4) <30 days in milk (DIM) had a 10-year mean of 14.57% (annual median ranged from 10.81 to 20%), decreasing to 8.10% (annual median ranged from 6.09 to 12.90%) from ≥30 to <60 DIM, with annual variation noted. Multivariable binomial mixed-effects regression models identified factors associated with an increased herd-level prevalence of high FPR including, in the <30 DIM model, months of February and March; genetic predicted transmitting ability (PTA) for Fat kg and an increase in herd percentage of primiparous cows by 10%. In the ≥30 to <60 DIM model, the month of February, a 10% increase in herd percentage of primiparous cows, a 10% increase in herd percentage of parity 4+ cows, and genetic PTA for Fat kg, were associated with increased herd-level prevalence of high FPR. In both models, higher-yielding herds were associated with reduced odds of increased herd-level prevalence of high FPR. These findings suggest that the greatest risk period for high herd-level prevalence of FPR in this system occurs during the spring housing period and shortly after turnout to grass. Higher-yielding herds appeared to have lower risk of NEB and genetic selection for milk Fat kg had an influence on herd-level prevalence of high FPR.

## 1. Introduction

The Irish dairy industry primarily comprises seasonal, spring-calving, pasture-based herds [1]. In such systems, reproductive management aims to achieve high conception rates within a limited timeframe, ensuring consistent herd size and a uniform calving pattern [2,3]. Compact seasonal-calving systems align herd peak lactation with the period of optimal grass growth, maximizing feed efficiency and milk productivity [4,5,6]. In Ireland, the majority of dairy cows calve between January and April with peak calving in February and March [7]. The achievement of optimal fertility performance in this seasonal system can be highly influenced by nutritional management and negative energy balance in the spring and early summer periods [8]. While pasture-based production is considered economically, environmentally, and animal-welfare friendly [9,10], achieving high dry matter intake (DMI) in spring can be challenging due to housing-related factors such as high stocking density, limited feed space, limited grass availability [11], or challenging grazing conditions due to high levels of precipitation [12]. Compared with total mixed ration (TMR) feeding, pasture-based diets typically yield lower DMI [13], increasing the risk of negative energy balance (NEB) in early lactation cows. In seasonal pasture-based systems, NEB can affect a large proportion of a herd simultaneously due to synchronized lactation stages and grazing constraints [14].

NEB in early lactation dairy cows can lead to an increase in production diseases such as clinical and subclinical ketosis (SCK) [15], displaced abomasum [16], metritis, endometritis, and mastitis [17,18], with subsequent negative effects on fertility performance [19,20], increased prevalence of non-infectious foot lameness [21,22], and reduced 305-day yield [23,24]. Monitoring of energy balance (EB) is vital for managing herd health and productivity. Commonly, blood metabolites, such as beta-hydroxybutyrate (BHB) and non-esterified fatty acids (NEFAs), are measured in early lactation [23,25], with high levels being associated with severe NEB [26] and established thresholds, predicting clinical and subclinical diseases such as ketosis [27,28]. However, alternative non-invasive options have also been investigated and proven to be reliable monitors of EB in dairy herds.

A non-invasive method, suitable for use in all types of dairy systems, is the indirect monitoring of EB using individual cow and/or bulk milk solids [29,30]. Milk composition data, especially high fat-to-protein ratio (FPR), is widely recognized as a reliable proxy indicator for NEB, as it reflects the metabolic state of cows under energy deficit [31]. A FPR > 1.4 or >1.5 [32,33] has been used in previous studies as a marker of NEB in early lactation cows. FPR has shown more reliable associations with EB in early lactation than individual milk fat or protein concentrations [34,35,36]. Owing to its correlation with NEB [36], FPR has been proposed as a method to diagnose SCK in dairy cows [37,38]. Duffield et al. [37] reported an optimal FPR threshold of >1.33 to diagnose SCK (BHB ≥ 1.2 mmol/L) in the first 65 days in milk (DIM) with a sensitivity (Se) and specificity (Sp) of 58% and 69%, respectively. Jenkins et al. [39] identified a higher FPR cut-off of >1.42 (AUC = 0.83; *p* < 0.001), yielding higher Se (92%) but lower Sp (65%), suitable for herd-level screening despite increased risk of false positives. A recent study by [30] investigated the use of FPR in Irish pasture-based herds to assess negative energy balance (NEB) at the individual animal level, finding a moderate correlation with energy balance indicators such as BHB concentrations and a potential link between FPR and early postpartum blood calcium levels. In a recent Northern Irish study by [33], FPR scores in milk (>1.5) were consistently associated with greater NEB, milk yields, body weight loss, and plasma NEFA concentrations. It was concluded that FPR can provide an indication of EB at a herd level [33].

Monitoring of individual cow milk solids and related herd-level energy status requires regular milk recording. By the end of 2023, 60% of Irish dairy herds participated in milk recording, encompassing 81% of cows being milk recorded at least once annually [40]. In Ireland, milk recording data is collected centrally by the Irish Cattle Breeding Federation (ICBF; Co. Cork, Ireland), which supports genetic improvement including the development and refinement of genetic breeding indices, such as the multi-trait single figure profit index Economic Breeding Index (EBI) [41]. Since 2022, EBI incorporates eight sub-indices/genetic predicted transmitting abilities (PTAs), including milk production (milk yield and solids yield), fertility, calving performance, beef carcass, cow maintenance, cow management, carbon emissions, and health [42]. As Irish farmers are primarily paid per kilogram of milk solids produced per cow, EBI is weighted accordingly with higher PTA cows producing more milk protein and fat, allowing farmers to focus breeding selection towards high solids producing cows [43]. EBI has been validated for milk yield, milk solids production [44], and fertility performance [45] under varying feeding strategies.

Since the abolition of the European Union (EU) milk quota in April 2015, Ireland has experienced increases in stocking density, milk yield, and milk solids output [46,47]. Genetic selection for higher milk yields in the past, without corresponding increases in DMI, resulted in adverse outcomes, including a higher risk of NEB and associated economic losses [48,49,50]. Monitoring dairy herd health is critical for enhancing productivity and welfare while simultaneously reducing greenhouse gas emissions [51]. Despite the importance of managing early lactation NEB in Irish seasonal-calving dairy herds, national herd-level data on prevalence, trends, and risk factors remain scarce.

Addressing these knowledge gaps, the objectives of this retrospective data-based observational study were to describe the herd-level prevalence of high FPR during early lactation in Irish spring-calving dairy herds, and to identify factors associated with herd-level prevalence of high FPR.

## 2. Materials and Methods

### 2.1. Ethical Approval

Ethical exception for the use of the data in this study was granted by the University College Dublin (UCD) Animal Research and Ethics Committee (AREC-E-24-2-Ryan).

### 2.2. Data

This desk-based study involved the analysis of milk-recording data from Irish seasonal, spring-calving dairy herds. Individual cow data from milk recording herds between 2014 and 2023, inclusively, were sourced from the Irish Cattle Breeding Federation (ICBF) national database (www.icbf.com). Milk test-day records included data on 305-day milk yield (kg); fat and protein yields (kg); and percentages, parity, calving date, EBI and its subindices (milk yield, fertility, fat and protein percentages, milk Fat kg, milk Protein kg), which are genetic measures of PTA for these performance; and milk solids components. For the purposes of this study, 305-day milk yield refers to predicted 305-day milk yield as calculated by ICBF using a test-day model [52]. Whilst all parities of cows were retained for analysis in this study, the proportions of parity 1 and parity 4+ cows were specifically used as additional independent variables in this analysis for two reasons: (i) as proxy measures of changing herd dynamics, e.g., herd expansion was assumed to be associated with an increased proportion of primiparous cows in the herd, and (ii) due to prior research showing that primiparous and older cows are at a higher risk of NEB [36,52]. Information on breed was not available in the research study dataset.

### 2.3. Data Cleaning and Analysis

Microsoft Excel [53] csv data files were obtained from ICBF. The initial dataset included 30,859,853 milk records from 19,396,494 unique cows in 12,875 unique herds spanning a 10-year period. Further data cleaning, visualization, and analysis were carried out exclusively in RStudio [54] version RStudio 2024.12.1+563 “Kousa Dogwood”. Outlier analysis and data edits were performed as follows: records with missing values for test-day (TD) milk yield (kg), TD milk fat (kg), TD milk protein (kg), EBI, herd identification, or test date were excluded. Test-day records with values exceeding ±3 standard deviations (SD) from the mean for TD milk yield (kg), TD milk fat yield (kg), and TD milk protein yield (kg) were omitted. Records from cows with more than 10 parities were removed. Days in milk (DIM) was calculated for all cows by subtracting the calving date from the milk-recording date, and records with DIM < 1 or >305 were omitted. This left 28,572,326 eligible individual cow milk records.

For subsequent herd-level data management, a “herd” was defined as all cows with the same unique herd identifier on each monthly test day across the dataset. Herds were required to have greater than 20 cows milk recorded on any test day for inclusion in the analysis. During the <30 DIM period, herds were required to have at least 10 cows with <30 DIM recorded on any test day. Similarly, herds were required to have at least 10 cows ≥30 DIM and <60 DIM recorded on any test day for analysis within that time window. Given that study objectives were centered on early-lactation milk solids analysis in spring-calving herds, the study data was restricted to milk recordings in the first 6 months of the year. Autumn-calving herds were excluded from the analysis, i.e., only milk recordings from January to June between 2014 and 2023 were included.

In this study, the fat-to-protein ratio was used as a proxy measure for herd-level NEB, and, as a result, a number of calculations were composed using the raw data. A high fat-to-protein ratio was defined as an individual cow test day FPR > 1.4, with herd-level prevalence then calculated as the sum of the individual cows with FPR > 1.4 divided by the number of cows per herd whose milk was recorded for both the <30 DIM and the ≥30 to <60 DIM time periods [23,34].

### 2.4. Statistical Analysis

Data cleaning, analysis, and visualization were carried out in RStudio [54] and the packages “dplyr” [55] “data.table” [56], and “tidyverse” [57]. Missing data was not imputed and was treated as an N/A. Preliminary descriptive statistics were performed on all variables to gain a basic understanding of the study population. For continuous variables, mean, median, and interquartile range (IQR) were calculated, with SD and range also calculated for herd 305-day-milk yield. For categorical variables, counts and percentages were calculated. Continuous data were first visualized using histograms to investigate if they were normally distributed.

### 2.5. Statistical Models

Two separate multivariable binomial mixed-effects regression models were created using “lme4” [58] to determine factors associated with early-lactation herd-level prevalence of high FPR (dependent variable) in study herds for both the <30 DIM and ≥30 to <60 DIM time periods.

The independent variables assessed for association in these models included the following: the number and proportion of first lactation (parity 1) cows; the number and proportion of fourth+ lactation (parity 4+) cows (while the analyzed dataset included all parities, these particular parities were added specifically as proxy measures of herd expansion and changing herd dynamics); mean 305-day-yield; median 305-day-yield; minimum and maximum 305-day-yield; range in 305-day-yield (calculated as maximum minus minimum); SD of 305-day-yield; 10th and 90th percentiles of 305-day-yield; month; mean of genetic (EBI) subindices, i.e., mean PTA for Protein kg, mean PTA for Fat kg; mean PTA for Milk kg; and the number of cows whose milk was recorded at each test day. Herd was treated as a random variable to account for potential farm-to-farm variation expected as a result of variations in management-, nutrition-, and infrastructure-related factors. The year was also treated as a random variable to account for variations in annual weather conditions, resultant impacts on the duration of housing in the post-parturient period, subsequent grass growth, and requirements for nutritional supplementations or alterations that could have biased the herd-level risk of high FPR across the study period. The multivariable models were built using backwards stepwise elimination. Variables with a *p*-value > 0.05 were sequentially removed, and only statistically significant variables were retained in the final model. Correlation between variables was evaluated using the Pearson correlation coefficient; if variables were highly correlated (r > 0.8), the model was run with each variable independently, and the Akaike Information Criterion (AIC) was used to identify the best model fit, resulting in the retention of the variable associated with the lowest AIC. Pseudo-R-squared and intraclass coefficients were estimated using the methods described proposed by Nakagawa (2017) [59] using the “performance” package. Model fit was further assessed based on visualization of calibration plots.

The herd-level prevalence of high FPR was assessed for the time periods <30 DIM and from ≥30 DIM and <60 DIM in study herds, with a separate model produced for each time window. Odds ratios (OR) were calculated, including 95% confidence intervals around them, to illustrate the strength of association of each independent variable predictor with herd-level prevalence of high FPR. Illustrative model predictions were produced to demonstrate the impact of the effect sizes on the outcome, i.e., herd-level prevalence of high FPR. Two-way interactions between milk yield variables (mean, range, and SD in 305-day-yield) and genetic PTA variables (mean EBI/PTA Fat and Milk kg) were assessed and interactions with a *p* < 0.05 are displayed in the Results.

## 3. Results

### 3.1. Descriptive Statistics

Descriptive statistics are summarized in Table 1. After removing missing values and outliers and data edits, the final dataset consisted of 327,257 milk recordings from 11,051 unique herds. The majority of calvings occurred in the first 100 days of the year, with the peak calving time occurring around mid-February. On average, the number of cows <60 DIM per milk-recording test day during the study period was 88 cows, increasing from 69.5 in 2014 to 100 in 2023. The number of herds whose milk was recorded per year increased from 6263 in 2014 to 9072 in 2023 with an average of 6942.9 over the ten-year period. The average number of herds whose milk was recorded increased by 34% from 2021 to 2023 in comparison to the first seven years of the study period. Across the study period, the mean herd 305-day-yield was 6591.97 kg, median 305-day-yield was 6601.78 kg, SD in 305-day-yield across all herds combined was 889.33 kg, and the range in 305-day-yield was 4063.62 kg. The SD and the range in 305-day-yield increased from 2014 to 2023. The average percentage of parity 1 cows per herd was 25.78% and 36.44% for parity 4+ cows. The proportions of primiparous and parity 4+ cows per herd varied between the first 5 years of the study and the second 5-year period. From 2014 to 2018, the average percentage of primiparous cows per herd was 27% and 33.97% for parity 4+ cows. From 2019 to 2023, the average percentage of primiparous cows decreased to 24.3%, and the average percentage of parity 4+ cows increased to 38.92% (Table 1). Average herd EBI subindex/genetic PTA for Milk kg, Protein kg, and Fat kg were 60.07 kg, 4.51 kg, and 5.34 kg, respectively, across the study period (Table 1). The EBI for dairy herds included in this study showed a consistent upward trend over the 10 years of the study period. The average herd genetic PTA for milk kg increased by 51% from 2014 to 2023, with the genetic herd PTA for both Protein kg and Fat kg increasing by approximately 200% (Table 1).

### 3.2. Herd-Level Prevalence of High FPR

**Figure 1:**
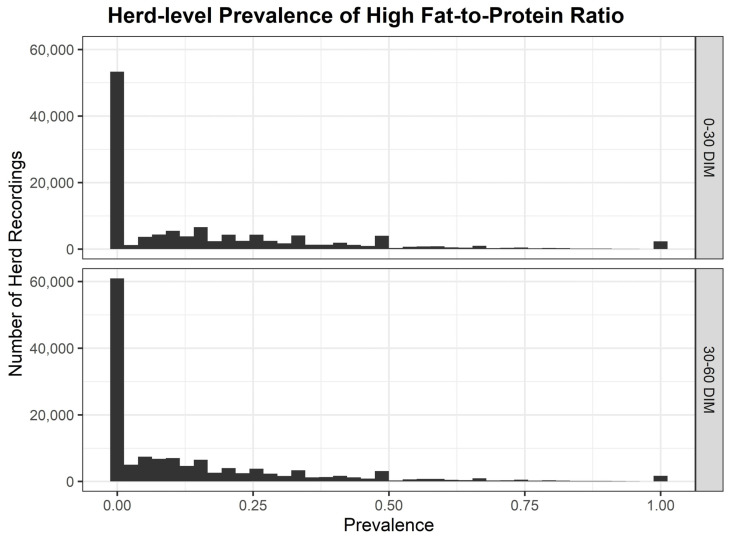
Herd-level prevalence of high fat-to-protein ratio <30 and ≥30 to <60 DIM across the 10-year study period.

The distribution of herd-level prevalence of high FPR from 2014 to 2023 is displayed in Figure 1. Median herd-level prevalence of high FPR had a 10-year mean of 14.57% <30 DIM and 8.10% from ≥30 to <60 DIM (Table 2). Annual variation in the herd-level prevalence of high FPR was evident, with 2018 emerging as a year with the most significant elevation, resulting in a median herd-level prevalence of high FPR of 20.0% <30 DIM and 12.90% from ≥30 to <60 DIM. There was a significant inter-herd variation in the herd-level prevalence of high FPR by year based on the breadth of the interquartile ranges (Table 2). IQRs varied from 21.77% in 2019 to 30.96% in 2018 <30 DIM, and from a low of 16.28% in 2019 to a high of 27.92% in 2018 from ≥30 to <60 DIM. The 75th percentile of herd-level prevalence of high FPR <30 DIM was highest in 2018 at 39.29% but was ≥25% across the 10-year study period.

### 3.3. Herd-Level Prevalence of High-Fat-to-Protein Ratio Models

#### 3.3.1. <30 DIM Model

The <30 DIM model looked at associations between herd-level prevalence of high FPR > 1.4, a proxy indicator of NEB, and the variables parity, month, 305-day-yield, EBI subindices (genetic PTA for Milk kg, Protein kg, and Fat kg), and number of cows per milk-recording test day. For the purposes of this multivariable binomial mixed-effects regression model, both herd and year were treated as random variables (Table 3).

For the <30 DIM model, the intraclass coefficients were 0.184 for the herd level and 0.004 for year, indicating that a significant proportion of the variance was at the herd level. The conditional pseudo-R-squared (including both fixed and random effects) was 0.243, and the marginal pseudo-R-squared (fixed effects only) was 0.068. Calibration plots demonstrated that mean observed high FPR prevalence was similar to the center of the prediction bin; however, there was significant variation among the observed.

**Figure 2:**
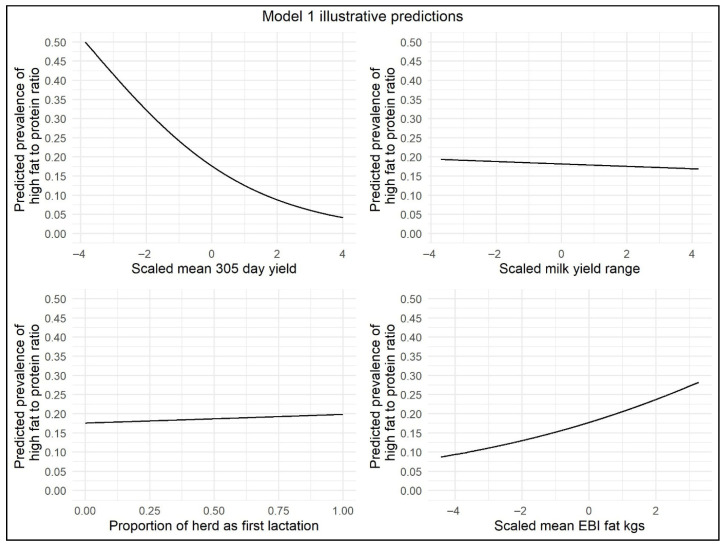
Illustrative <30 DIM model predictions demonstrating the impact of scaled mean 305-day-yield, scaled range in 305-day-yield, proportion of parity 1 cows per herd, and scaled mean EBI/PTA Fat kg on herd-level prevalence of high FPR.

This model showed that there was an increased odds (OR = 1.015) of herd-level prevalence of high FPR <30 DIM in herds with each 10% increase in percentage of parity 1 cows, i.e., herds with a younger age profile based on the percentage of parity 1 cows had a slightly increased risk of NEB. The months of February and March were associated with increased odds (OR = 1.237 and OR = 1.140, respectively) of the herd-level prevalence of high FPR. In contrast, in April, May, and June, the odds of herd-level prevalence of high FPR were decreased (OR = 0.636, OR = 0.455, and OR = 0.476, respectively). For the variables herd range and mean 305-day-yield, the odds of herd-level prevalence of high FPR were significantly decreased with each 1000 kg increase in milk yield (OR = 0.98 and OR = 0.636, respectively). There was an increase in the odds of herd-level prevalence of high FPR in herds with an increased genetic PTA for Fat kg (OR = 1.058). The number of cows per milk-recording test day was retained as a significant variable in this model (*p* < 0.05), but an increase of 10 cows recorded had minimal effect on the odds of herd-level prevalence of high FPR (OR = 0.994). Illustrative model predictions have been included to demonstrate the impact of the effect sizes on the model outcome (Figure 2).

A negative interaction was found between mean 305-day-yield and mean EBI/PTA Fat kg, i.e., as the milk yield increased, the positive effect of EBI/PTA Fat kg on the odds of herd-level prevalence of high FPR became smaller. Therefore, in this study population, a high milk yield reduced the odds of herd-level prevalence of high FPR more sharply in herds that were genetically selected for higher fat yield compared to herds genetically selected for lower fat yield. A positive interaction was found between range in 305-day-yield and mean EBI/PTA Fat kg. In this study population, herds with greater variability in 305-day-yield were more likely to have higher herd-level prevalence of high FPR if genetically selected for higher fat yield compared to herds with lower genetic selection for fat yield.

#### 3.3.2. ≥30 to <60 DIM Model

For the ≥30 to <60 DIM model, the intraclass coefficients were 0.215 and 0.009 for herd- and year-level random effects, respectively. The conditional pseudo-R-squared (including both fixed and random effects) was 0.303, and the marginal pseudo-R-squared (fixed effects only) was 0.101. Calibration plots demonstrated that the mean observed high FPR prevalence was similar to the center of the prediction bin for lower predicted values (<0.4). At higher predicted values (>0.4), it tended to be lower than the observed values. However, there was significant variation among the observed prevalences.

**Figure 3:**
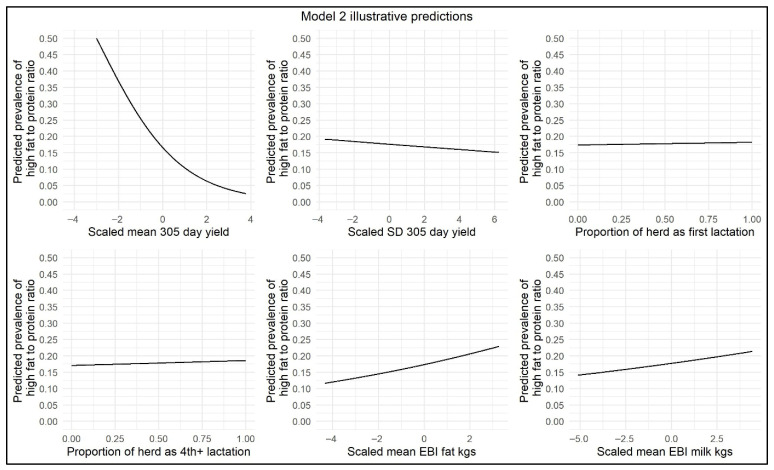
Illustrative ≥30 to <60 DIM model predictions demonstrating the impact of scaled mean 305-day-yield, scaled SD in 305-day-yield, proportion of parity 1 cows per herd, proportion of parity 4+ cows per herd, scaled mean EBI/PTA Fat kg, and scaled mean EBI/PTA Milk kg on herd-level prevalence of high FPR.

The results of the ≥30 to <60 DIM model are displayed in Table 3, and illustrative model predictions have been included to demonstrate the impact of the effect sizes on the model outcome (Figure 3). This model showed that, for each 10% increase in percentage of parity 4+ cows, there was a slight increase in the odds (OR = 1.008) of herd-level prevalence of high FPR during this time window, suggestive of an increased risk of NEB in herds with an older herd age profile. The odds of herd-level prevalence of high FPR were marginally increased with each 10% increase in the percentage of parity 1 cows (OR = 1.007). In the month of February, the odds of herd-level prevalence of high FPR were significantly increased (OR = 1.272), suggesting a greater risk of NEB during that month. The odds of herd-level prevalence of high FPR were significantly decreased from the months of March to June (OR = 0.904, 0.434, 0.302, and 0.357, respectively). With respect to SD and the mean 305-day-yield, with each increase of 1000 kg in 305-day-yield, there was a significant decrease in the odds of herd-level prevalence of high FPR (OR = 0.857 and OR = 0.543, respectively). With increasing genetic PTA for Fat kg, there was a greater odds of increased herd-level prevalence of high FPR (OR = 1.040). Mean genetic PTA for Milk kg was retained as a significant variable (*p* < 0.05) but had no effect on the odds of herd-level prevalence of high FPR (OR = 1.000). The number of cows per milk-recording test day was also retained as a significant variable in this model. With each increase of 10 cows per milk-recording test day, the odds of the herd-level prevalence of high FPR were marginally reduced (OR = 0.985).

Similar to the <30 DIM model, a negative interaction was found in the ≥30 to <60 DIM model between mean 305-day-yield and mean EBI/PTA Fat kg. There were positive interactions between mean 305-day-yield and mean EBI/PTA Milk kg and between SD in 305-day-yield and mean EBI/PTA Milk kg. This suggests that in higher-yielding herds, changes in EBI/PTA Milk kg were associated with smaller changes in herd-level prevalence of high FPR compared to lower-yielding herds.

## 4. Discussion

The large scale of this dataset, combined with its focus on Ireland’s pasture-based and seasonal-calving system, distinguishes this research from prior studies that have predominantly targeted more controlled or TMR feeding systems [28,60]. Also, this study adds to the small number of previous publications, which have used FPR as a proxy indicator of NEB [30,35] in herds on the island of Ireland. Moreover, the integration of herd-level parameters, including parity distributions, month, yield parameters, and genetic PTA’s, facilitated a detailed herd-level analysis of factors associated with the multifaceted nature of FPR in the Irish spring-calving system, where environmental, management, and genetic factors interplay [11,46,61].

### 4.1. Herd-Level Prevalence of High FPR in Early Lactation

It has been shown that the herd-level prevalence of high FPR is a relatively good herd-level indicator of energy status in early lactation [33,62,63], justifying its use in this study. Frequently, cut offs of ≥1.4 or >1.5 have been used as thresholds for high FPR in previous studies and have been associated with ketosis and NEB [64,65,66,67]. A 2015 study in a U.S. indoor dairy herd found that using an individual cow FPR cut-off of >1.42 yielded a Se of 92% and Sp of 65% in relation to herd-level screening for NEB [39]. This apparent lack of specificity does mean that there is an increased risk of misclassification of energy balance status in dairy herds when using FPR, which may have influenced the results of our study, resulting in more herds being falsely classified as having NEB.

The median herd-level prevalence of high FPR (>1.4) in this study had a 10-year mean of 14.57% <30 DIM and 8.10% ≥30 to <60 DIM from 2014 to 2023, with the 75th percentile of annual herd-level prevalence of high FPR consistently ≥25% <30 DIM. A monitoring threshold of <10% for herd-level prevalence of high FPR (>1.5) has been proposed in previous research [23,32,38], with the results of this study suggesting that NEB in the first month of lactation has been an important problem in the Irish seasonal spring-calving system. However, when cows were calved >30 DIM, there was a lower herd-level prevalence of high FPR, suggesting a decreased risk of NEB. Although they used a higher threshold for FPR than the >1.4 threshold used in the present study, a prior Irish study by Cabezas-Garcia et al. [33] found similar results, with 17.2% of cows having a high FPR > 1.5 in the first four weeks of lactation. There are a limited number of studies published where energy status was monitored using herd-level prevalence of high FPR in seasonal grass-based systems, with the majority of publications referring to high-yielding indoor herds on TMR diets [32,62]. An Italian study by Toni et al. [60] found an incidence of around 30% of cows with a high FPR (>2.0) on day 7 postpartum in indoor high-yielding herds (305-day-corrected-yield of approximately 9500 L). These authors also reported that cows with an FPR > 2.0 showed increased incidences of retained fetal membranes, left displaced abomasum, clinical metritis, and endometritis [60]. In a Canadian study, there was a 44% incidence of high FPR > 1.42 (sensitivity = 92%; specificity = 65%) in lactating cows between 8 and 30 DIM [39]. While there are many publications linking thresholds for high FPR, as a proxy for NEB/SCK, with common metabolic and production diseases in the transition and post-parturient periods [30,68,69], the prevalence of high FPR at herd or animal level is not described in many studies [38,62,66]. Other studies have described the prevalence of SCK in dairy herds using the measurement of blood BHB. For example, one study from New Zealand found an SCK prevalence of 14.3% in pasture-based grazing dairy herds using a BHB-threshold of ≥1.4 mmol/L [70]. Using BHBs, the prevalence of SCK has generally been found to be lower in grass-based systems [23,30,71], with several studies observing a prevalence of 20–30% for predominantly indoor systems across Europe [28,72,73].

Annual variation in the herd-level prevalence of high FPR was evident with 2018 emerging as a year with the most significant elevation resulting in a median herd-level prevalence of high FPR of 20.0% <30 DIM, and a median of 12.9% ≥30 to <60 DIM. In 2018, Ireland experienced severe weather conditions with a significant cold period in late February and early March, often called the “Beast from the East”, with accompanying heavy snow fall and conditions unfavorable for grass growth [74]. As a result, due to a lack of grass growth and a significant fodder shortage, it is likely this resulted in an increased herd-level prevalence of high FPR in early lactation. Grazing systems are particularly exposed to challenges resulting from prevailing weather conditions. In an Irish context, it can be difficult to achieve high levels of DMI in spring caused by low grass availability [11] or demanding grazing conditions due to high levels of precipitation [12].

### 4.2. Risk Factors Associated with Herd-Level Prevalence of High FPR

The risk factors associated with herd-level prevalence of high FPR in the current study were month of the year, 305-day-yield parameters, genetic PTA for Fat and Milk kg, percentage of parity 1 and parity 4+ cows, and number of cows per milk-recording test day.

#### 4.2.1. Month of the Year

An important finding of this study was the effect of the month of the year on the herd-level prevalence of high FPR. In the context of herd-level prevalence of high FPR as a proxy indicator of NEB, this study showed that early-lactation cows were at a greater risk of NEB during the months of February and March compared to the later spring/early summer period of April and May. This likely relates to the structure of Ireland’s compact seasonal-calving system, where peak calving occurs in February and most cows are turned out to grass by mid-March to align peak lactation with optimal grass growth, thereby maximizing feed efficiency and milk productivity [4,5,6]. Cows are usually housed indoors and fed silage until the middle of March in Ireland [75], meaning that increased herd-level prevalence of high FPR <30 DIM in early lactation is essentially an issue of the housing period and the immediate period following turnout to grass. Unfortunately, the lack of herd-level data on feed space, silage quality, transition cow feeding, and grazing conditions is a limitation of this study and constrains causal inference in relation to these factors. The authors suggest further research in this area, potentially integrating on-farm management surveys or remote sensing of grass growth.

Housing-related challenges in the Irish dairy system include limited feed and cubicle space and milking parlor bottlenecks during the time of greatest stocking density, i.e., peak calving. This can lead to prolonged standing times and reduced opportunities for rumination and feeding [76,77], factors associated with an increased risk of clinical or subclinical ketosis [78]. An Irish study by Crossley et al. [79] found that average feed space (0.52 m/cow) during the housing period on farms was below the recommended target (0.6 to 0.75 m/cow) [80]. The DMI of silage can be negatively affected by reduced feeding space and a competitive feeding environment [81]. Additionally, low ground temperatures restrict grass growth in early spring, limiting availability during the early post-turnout period [82,83]. Greater availability of good quality grass covers, with grass having superior feeding quality due to its higher digestibility and crude protein (CP) content compared to grass silage [84], allied to the lack of housing-related factors, may explain the reduced risk of NEB observed in April and May when grass growth peaks. Another important influence on milk fat percentage, which is very relevant in the Irish spring-calving system, is the well-documented issue of milk fat depression (MFD) or low milk fat syndrome, which is defined as reduced milk fat yield in the presence of normal or expected milk yield or yield of other milk components, such as milk protein [85]. In a recent Irish study, findings by Carty et al. (2017) [85] showed a consistently high prevalence of MFD in April and May each year across an 11-year study period up to 2014. It is accepted that high concentrations of polyunsaturated fatty acids in grass may precipitate MFD [85], meaning that early-lactation dairy cows with a predominantly grass-based diet could be expected to have lower milk fat percentages and, as a result, a lower herd-level prevalence of high FPR from April onwards. It is the authors’ opinion that the use of herd-level prevalence of high FPR is not likely a reliable indicator of energy status from the end of April onwards.

#### 4.2.2. 305-Day-Yield

In 2014, the mean 305-day-yield was 6561 kg, and, although it peaked at 6713 kg in 2021, there was a subsequent decrease to 6444 kg in 2023. Data from Teagasc, the Agriculture and Food Development Authority, showed an increase in yield from 5170 L in 2014 to 5781 L in 2021 and a reduction to 5474 L in 2023 per cow [86]. The decline in mean 305-day-yield since 2021 may be reflective of a change in cow type and genetic selection towards milk solids rather than milk yield. The difference in yield between the Teagasc data and this study based on ICBF data is likely reflective of increased herd size overall in milk recording herds and bias towards greater yield.

This study found that a higher range and mean 305-day-milk yield were associated with reduced herd-level prevalence of high FPR, suggesting that NEB was less prevalent in higher-yielding herds. This was an interesting result and contrary to previous study findings, indicating that higher-yielding cows were at greater risk of NEB [32,87,88,89]. However, those studies were mainly carried out in indoor feeding systems, where the milk yield would have been substantially greater in comparison to our study herds, where the maximum annual 305-day-yields were <9000 kg. Previous Irish research has shown that milk-recording herds tend to have higher milk yields, are better managed, and are run by farmers with a higher level of agricultural education than non-milk-recording herds [90,91]. Although detailed data on transition and early-lactation cow management of study herds were not available, similar trends within this cohort of milk-recording herds towards better management in higher-yielding herds may be valid. The finding that a higher 305-day-yield was associated with lower odds of herd-level prevalence of high FPR, i.e., lower odds of NEB, has highlighted an important knowledge gap, and further research is warranted to answer the question of why higher yielding herds appear to be at lower risk of NEB in the Irish spring-calving system. It is possible that selection for milk solids rather than volume and genetic adaptation to pasture might decouple yield from NEB risk. Another hypothesis may relate to the supposition that lower-yielding herds may have superior fertility performance in the Irish grass-based system resulting in tighter calving patterns peaking in February. Earlier compact calving may be contributing to increased housing, management, and metabolic stress during the months of February and March. Increased herd-level prevalence of high FPR in February and March, consistent with peak calving in Ireland, was a significant finding of this study. Finally, an additional explanation could be that higher-yielding herds may have more mitigation for NEB, given the higher nutritional inputs and dietary management requirements compared to lower-yielding herds, which are likely more susceptible to nutritional imbalances due to factors, such as lack of feed space or grass shortages.

#### 4.2.3. Genetic PTA

Despite a national EBI base change by ICBF in September 2016 that scaled back overall genetic index values [92], an increase in overall population genetic PTA was evident, with substantial increases in overall EBI, as well as genetic PTA for Milk kg, Fat kg, and Protein kg, across the 10-year study period. This resulted in an increase of over 200% in both the average herd genetic PTA for Fat kg and Protein kg from 2014 to 2023, with the fat PTA showing the largest increase. These findings are consistent with national data from the National Milk Agency, which showed that the average Irish annual herd milk fat (3.99% to 4.30%), and protein percentage (3.43% to 3.52%) increased over the 10-year period [93]. In our study, the genetic PTA for Milk kg increased by 150%, despite no significant upward trend in the 305-day-yield from 2014 to 2023.

The genetic profile of Irish dairy herds has moved towards cows selected for higher milk solids [41]. While breed information was not available for inclusion in this study, this was partially addressed through the use of EBI/genetic PTA for Protein kg and Fat kg, which reflect genetic potential for increased milk solids production. Additionally, the use of FPR as an indicator of energy balance offset the limitation of lack of breed related data, as it is more reliable than alternatives, such as milk protein percentage, when dealing with herds that contain a proportion of Jersey or crossbred genetics, although higher cut offs may need to be used [94,95]. In 2023, the Department of Agriculture AIM Bovine Statistics Report indicated that Jersey cows represented approximately 4.75% of the Irish national dairy herd [96]. Whilst we included genetic indices as part of our analysis, we did not have access to the specific breed of the animals. It is possible that some breed effects, e.g., in Jersey animals, may have had greater effects on milk solids composition beyond that implied by their genetic index. As a result, the authors recommend the inclusion of breed data in future work when possible.

This study found that higher genetic PTA for Fat kg was associated with a slightly greater odds of increased herd-level prevalence of high FPR, i.e., a marginally greater risk of early lactation NEB. Cows with higher genetic PTA for Fat kg would likely be producing higher levels of Fat kg and, as a result, would more easily reach the high FPR threshold of >1.4 if nutritional management was suboptimal. Heifers or first lactation animals under the prevailing breeding strategies of the Irish seasonal system are expected to have more advanced genetic merit, including higher PTA for Fat kg [97]. Therefore, there may be some confounding of risk in herds with a greater proportion of primiparous cows. A possible explanation for the positive association between genetic PTA for Fat kg and an increase in herd-level prevalence of high FPR is the consequent energy demand associated with producing greater amounts and percentages of milk fat. An Irish study investigating the relationship between genetics and early-lactation feeding management found that cows with low EBI fertility subindex/genetic PTA for fertility and a high PTA for milk yield and milk solids (fat and protein) (LFHM) had a tendency for greater NEB than cows with high genetic PTA for fertility and low PTA for milk yield and milk solids (HFLM) [98]. The LFHM cows produced more milk and milk solids overall [61]. Another Irish single herd study comparing high-yielding pedigree Holsteins (92% Holstein) with Irish Holstein-Friesian (HF) cows (52% Holstein) found that the HF cows had higher milk solids production and less severe NEB in early lactation [99]. However, the herd-level approach and size of our study population validates the finding of increased herd-level prevalence of high FPR in Irish dairy herds with higher PTA for Fat kg.

#### 4.2.4. Percentage of Parity 1 and Parity 4+ Cows

This study showed that a higher proportion of parity 1 and/or parity 4+ cows in Irish dairy herds was associated with a marginally increased herd-level prevalence of high FPR, indicating a slightly higher risk of NEB. Primiparous cows typically have lower DMI than multiparous animals, in part due to smaller rumen capacity [14,100,101,102]. Additionally, first-lactation animals often face increased metabolic and social stress during the transition period, as they adapt to lactation while competing with older cows for feed, cubicle, and water access [103,104,105]. Following milk quota removal in 2015, rapid herd expansion occurred across many Irish farms, with new entrants transitioning from other sectors into dairying [106]. Our study observed a greater proportion of primiparous cows in study herds between 2014 and 2018, coinciding with this expansion period [106]. The finding of a significant positive association between herd-level prevalence of high FPR and a higher proportion of parity 1 cows suggests that there may be a greater risk of NEB in dairy herds under expansion. In contrast, the second half of this study showed a decline in the percentage of primiparous cows and an increase in percentage of parity 4+ cows, possibly reflecting a reduction in herd size expansion and stabilization of cow numbers [107]. Such herd growth often introduces infrastructural constraints, including increased stocking density, limited feed, and cubicle space.

The slightly increased NEB risk in primiparous cows may also reflect suboptimal heifer management in Irish dairy herds. Heifers calving in either excessive or suboptimal body condition are known to be at increased NEB risk post-calving [108,109]. It has to be noted that the majority of studies which looked at risk factors for early lactation NEB, including the impact of parity, have been conducted in indoor systems with only limited data available for a grazing scenario. Some research has indicated that older cows are also at increased risk of NEB [60,88,110]. Buttchereit et al. [36] reported higher FPR and more severe energy deficits in cows of parity 3+ compared to first- or second-lactation cows in early lactation. Similarly, a Canadian study found a higher prevalence of SCK with increasing parity based on test day milk fat and milk protein [37]. In this study, parity had only a minor association with herd-level prevalence of high FPR compared to other variables such as month or 305-day-yield.

#### 4.2.5. Number of Cows per Milk-Recording Test Day

Over the 10-year study period, the average number of cows with milk recorded per test day within the first 60 DIM increased from 69.6 in 2014 to 100 in 2023. This trend likely reflected herd expansion before and following the EU milk quota abolition in 2015 [111], a pattern also observed in other European countries [112]. Our study observed a 37% increase in the number of herds performing milk recording from 2020 to 2023. An Irish study by Balaine [91] reported that milk-recording herds tended to have larger herd size and higher milk yields. The increase in milk recording may reflect the implementation of EU regulation 2019/6 on Veterinary Medicines [113], which introduced stricter prescribing requirements for antibiotics, including cow-level data to support selective dry cow therapy [114]. Increased uptake may also be attributed to a broader industry focus on genetic improvement, milk quality, and general herd performance [92,115].

While the number of cows per milk-recording test day remained significant in the multivariable models, the association was weak, suggesting that herd size was not a major factor influencing the risk of NEB in Irish dairy herds. There have been similar findings in other studies in the UK and Canada where herd size has not been found to have a significant impact on the risk of NEB or SCK in dairy herds [28,116].

### 4.3. Practical Implications

In relation to guidance for farmers and advisory services, our study findings suggest that there is a greater risk of NEB in lower-yielding herds, herds with a higher percentage of parity 1 and parity 4+ cows, and herds with higher PTA for milk Fat kg during the months of February and March in the Irish spring-calving dairy system. Farmers and herd health advisors should be aware of these risks and should aim to identify higher risk herds with a view to closer monitoring of transition and postparturient cow health and energy balance status. Regular milk recording during the months of February and March, which is relatively uncommon currently due to time and labor constraints, should be recommended in order to provide data during the highest-risk period. Following identification of high-risk herds, additional on-farm factors should be assessed, including stocking density, feed space, dietary sufficiency, and grass availability. Better preventative strategies can reduce disease and antimicrobial usage and mitigate losses and culling, thereby leading to improvements in animal welfare and reductions in greenhouse gas emissions.

### 4.4. Limitations

The use of herd-level prevalence of high FPR has limitations when used as the sole measure of energy balance in a population of dairy cattle. As mentioned previously, this proxy measure is limited by its lower sensitivity and specificity in comparison to alternative measures, such as BCS and measurement of BHB concentrations [28,39]. It is recommended that future research should be carried out to validate the FPR > 1.4 threshold under Irish conditions, through direct comparison with BHB or NEFA concentrations, as it may improve diagnostic accuracy. Additionally, housing- and management-related factors, which impact at the herd-level, were outside the scope of this study, and information on breed was not available. While our study found an association between genetic PTA for Fat kg and herd-level prevalence of high FPR, it should be acknowledged that milk composition is also influenced by non-genetic factors, including diet or milking frequency [117,118]. However, the advantages of using herd-level prevalence of high FPR as a valuable and practical herd screening tool have been outlined previously. Approximately two-thirds of Irish dairy farmers regularly participate in milk recording [119]. However, due to the seasonal nature of production, the number of farmers recording data varies each month, with around 63.8% of cows recorded in the first 60 DIM in 2020/2021 [120]. As a result, the dataset and findings from the current study are representative of this specific biased subset of herds, which may affect the external validity of these study findings. Given that milk-recording herds are often more progressive herds [91], it could be argued that the true herd-level prevalence of high FPR across all Irish dairy herds may be even higher. It is common for Irish dairy farmers to carry out 4 to 6 milk recordings per year, of which 2 to 3 at most would occur from January to June. Finally, external factors, such as fluctuations in grass growth, quality, weather patterns, and feeding and housing management may influence herd-level prevalence of high FPR. As mentioned previously, it must be acknowledged that high concentrations of polyunsaturated fatty acids in grass, and subsequent MFD, may have limited the sensitivity of herd-level prevalence of high FPR as a proxy indicator of NEB in this study from late April onwards when grass is the primary component of the dairy cow diet. However, MFD was unlikely to be an issue in the months of February and March and likely had a limited overall effect on study findings. The lack of dietary, housing, and grazing data in this study limited the ability to assess the influence of these factors on study findings.

## 5. Conclusions

This large data-based study provides valuable information on the herd-level prevalence of high FPR in Irish seasonal spring-calving dairy herds, as well as risk factors associated with this proxy-measure of NEB. Herd-level prevalence of high FPR was highest in the first 30 DIM and lower between ≥30 and <60 DIM. There was a strong association between the herd-level prevalence of high FPR and the month of the year, with the highest odds occurring in February and March, which may reflect an association with housing and transition management in early-lactation cows before and shortly after turnout to grass. A higher 305-day-yield was associated with a lower herd-level prevalence of high FPR in this study, which is a significant finding for the Irish spring-calving dairy system. While explanatory theories including better management in higher-yielding herds, more compact and earlier calving in lower yielding herds, and selection for milk solids rather than volume together with genetic adaptation to pasture, have been proposed, further research is required to explain this finding. In this study, herds with higher genetic PTA for Fat kg had a higher odds of increased herd-level prevalence of high FPR. The association between parity and herd-level prevalence of high FPR was relatively minor in this study, with a higher percentage of first lactation and parity 4+ cows associated with a slightly increased herd-level prevalence of high FPR in early lactation. Understanding risk factors associated with herd-level prevalence of high FPR (early lactation NEB) is essential for improving herd health, productivity, and supporting the sustainability of Ireland’s expanding dairy sector.

## Figures and Tables

**Table 1:** Herd-level descriptive statistics, including mean number of cows per milk-recording test day, parity percentage, 305-day-yield parameters, and genetic PTA (EBI) during early lactation (0 to 60 DIM) across the 10-year study period from 2014 to 2023.

Year	nREC	%Parity1	%Parity4	Mean305 (kg)	Median305 (kg)	Min305 (kg)	Max305 (kg)	SD305 (kg)	Range305 (kg)	Mean EBI/PTA Milk kg	Mean EBI/PTA Prot kg	Mean EBI/PTA Fat kg
2014	69.56	27.7%	32.6%	6560.72	6550.20	4670.33	8536.70	863.07	3866.37	51.82	2.31	2.68
2015	75.72	29.2%	32.3%	6538.40	6528.33	4612.15	8540.51	873.04	3928.36	55.36	2.78	3.34
2016	81.20	27.0%	33.7%	6590.46	6585.60	4625.67	8625.48	882.91	3999.81	63.21	3.33	4.02
2017	85.13	25.9%	34.8%	6598.98	6599.32	4623.48	8599.65	865.24	3976.17	66.20	3.82	4.63
2018	89.51	25.8%	36.4%	6487.46	6495.09	4488.90	8512.23	877.19	4023.33	72.70	4.40	5.21
2019	90.31	24.2%	38.2%	6772.22	6799.40	4683.00	8794.78	895.64	4111.79	71.21	4.77	5.67
2020	97.43	24.6%	38.4%	6643.95	6662.52	4500.01	8753.88	925.04	4253.88	69.06	5.18	6.14
2021	96.90	24.2%	39.2%	6712.76	6739.94	4603.58	8754.09	902.17	4150.51	69.86	5.61	6.60
2022	98.64	23.9%	39.5%	6570.67	6594.03	4472.19	8647.43	907.58	4175.24	72.92	6.14	7.20
2023	100.05	24.7%	39.4%	6444.08	6463.46	4379.45	8530.24	901.45	4150.79	78.36	6.80	7.98
Average	88.45	25.7%	36.4%	6591.97	6601.79	4565.87	8629.50	889.33	4063.62	67.07	4.51	5.35

nREC = Mean number of cows per milk-recording test day; %Parity1 = percentage of parity 1 cows; %Parity4 = percentage of parity 4+ cows; mean305 = mean 305-day milk yield (kg); median305 = median 305-day milk yield (kg); min305 = minimum 305-day milk yield (kg); max305 = maximum 305-day milk yield (kg); SD305 = standard deviation of 305-day milk yield (kg); range = range in 305-day milk yield (kg); Mean EBI/PTA Milk kg = mean predicted transmitting ability for Milk KG; Mean EBI/PTA Prot kg = mean predicted transmitting ability for Protein KG; Mean EBI/PTA Fat kg = mean predicted transmitting ability for Fat KG subindex.

**Table 2:** Median herd-level prevalence of high fat-to-protein ratio (FPR) during early lactation in study herds from 2014 to 2023.

Year	Median Prevalence FPR 30	IQR Prevalence FPR 30	Median Prevalence FPR 3060	IQR Prevalence FPR 3060
2014	13.04%	22.92% (4.35–27.27%)	7.23%	18.75% (0.00–18.75%)
2015	13.33%	24.07% (4.76–28.83%)	6.25%	16.67% (0.00–16.67%)
2016	18.75%	29.71% (7.69–37.40%)	10.00%	27.27% (0.00–27.27%)
2017	14.29%	25.45% (4.55–30.00%)	7.14%	19.36% (0.00–19.36%)
2018	20.00%	30.96% (8.33–39.29%)	12.90%	27.92% (3.33–31.25%)
2019	10.81%	21.77% (3.23–25.00%)	6.09%	16.28% (0.00–16.28%)
2020	12.50%	22.88% (4.55–27.43%)	6.67%	17.74% (0.00–17.74%)
2021	14.29%	23.01% (5.56–28.57%)	8.00%	20.00% (0.00–20.00%)
2022	14.29%	23.91% (5.26–29.17%)	8.33%	20.00% (0.00–20.00%)
2023	16.75%	26.19% (7.14–33.33%)	10.74%	21.55% (3.45–25.00%)
Mean	14.57%	25.01% (5.33–30.34%)	8.10%	20.08% (0.68–20.76%)

Median Prevalence FPR 30 = median prevalence of high FPR <30 DIM; IQR Prevalence FPR 30 = interquartile range of high FPR < 30 DIM; Median Prevalence FPR 3060 = median prevalence of high FPR ≥ 30 to <60 DIM; IQR Prevalence FPR 3060 = interquartile range (25th–75th percentile) of high FPR ≥ 30 to <60 DIM.

**Table 3:** Multivariable binomial mixed-effects regression models looking at factors associated with herd-level prevalence of high fat-to-protein ratio <30 DIM and ≥30 to <60 DIM.

	<30 DIM					≥30 to <60 DIM				
Variable	Estimate	SE	*p*-Value	OR	95% CI	Estimate	SE	*p*-Value	OR	95% CI
%Parity4+ (10%) ^1^						0.008	0.003	0.003	1.008	1.003–1.014
%Parity1 (10%) ^1^	0.015	0.003	<0.001	1.015	1.010–1.020	0.007	0.003	<0.001	1.007	1.001–1.012
Month February	0.213	0.020	<0.001	1.237	1.199–1.276	0.240	0.024	<0.001	1.272	1.225–1.318
Month March	0.131	0.020	<0.001	1.140	1.100–1.179	−0.101	0.022	<0.001	0.904	0.861–0.948
Month April	−0.452	0.021	<0.001	0.636	0.596–0.677	−0.835	0.022	<0.001	0.434	0.390–0.478
Month May	−0.786	0.024	<0.001	0.455	0.409–0.502	−1.198	0.023	<0.001	0.302	0.257–0.347
Month June	−0.741	0.038	<0.001	0.476	0.402–0.551	−1.029	0.026	<0.001	0.357	0.307–0.408
Mean 305-day-yield (1000 kg) ^2^	−0.453	0.006	<0.001	0.636	0.624–0.647	−0.610	0.005	<0.001	0.543	0.533–0.553
Range 305-day-yield (1000 kg) ^2^	−0.022	0.004	<0.001	0.978	0.971–0.985					
SD 305-day-yield (1000 kg) ^2^						−0.155	0.020	<0.001	0.857	0.817–0.896
Mean EBI/PTA Fat kg	0.056	0.002	<0.001	1.058	1.054–1.062	0.039	0.002	<0.001	1.040	1.036–1.044
Mean EBI/PTA Milk kg						0.000	0.000	<0.001	1.000	1.000–1.001
No. of cows recorded (10 cows) ^3^	−0.006	0.001	<0.001	0.994	0.993–0.996	−0.015	0.001	<0.001	0.985	0.984–0.986
Mean 305-day-yield × Mean EBI/PTA Fat kg	−0.054		<0.001			−0.042		<0.001		
Range 305-day-yield × Mean EBI/PTA Fat kg	0.015		<0.001							
Mean 305-day-yield × Mean EBI/PTA Milk kg						0.045		<0.001		
SD 305-day-yield × Mean EBI/PTA Milk kg						0.049		<0.001		

SE = standard error; OR = odds ratio; 95% CI = 95% confidence interval; ^1^ = model estimate for a 10% increase in parity 1 and parity 4+ cows; ^2^ = model estimate for an increase in 1000 kg 305-day-yield; ^3^ = model estimate for an increase in 10 cows per milk recording.

## Data Availability

The original contributions presented in this study are included in the article. Further inquiries can be directed to the corresponding author.

## Abbreviations

The following abbreviations are used in this manuscript:

AIC = Akaike information criterion; EB = energy balance; EBI = Economic Breeding Index; EU = European Union; FPR = fat to protein ratio; HFLM = high EBI fertility subindex and low PTA for milk yield and milk solids; ICBF = Irish Cattle Breeding Federation; IQR = interquartile range; LFHM = low EBI fertility subindex and high PTA for milk yield and milk solids; NEB = negative energy balance; NEFA = non-esterified fatty acids; OR = odds ratio; PTA = predicted transmitting ability; SCK = subclinical ketosis; SD = standard deviation; Se = sensitivity; Sp = specificity; TD = test day
